# Effects of Respiratory Training on Pulmonary Function, Cough, and Functional Independence in Patients with Amyotrophic Lateral Sclerosis

**DOI:** 10.3390/neurolint16060101

**Published:** 2024-11-01

**Authors:** Eleonora Magni, Anja Hochsprung, Rocío Cáceres-Matos, Manuel Pabón-Carrasco, Beatriz Heredia-Camacho, Ignacio Solís-Marcos, Carlos Luque-Moreno

**Affiliations:** 1 Faculty of Nursing, Physiotherapy and Podiatry, University of Seville, Avenzoar 8 Street, 41008 Seville, Spain; magni@us.es (E.M.); beahercam2@alum.us.es (B.H.-C.); carloslm@us.es (C.L.-M.); 2 Research Group CTS-969: Care Innovation and Health Determinants, Avenzoar 8 Street, 41008 Seville, Spain; 3 Virgen Macarena University Hospital, Dr. Fedriani s/n, 41008 Seville, Spain; ahalcalag@gmail.com; 4 Research Group CTS-1050: Complex Care, Chronicity and Health Outcomes, Avenzoar 8 Street, 41008 Seville, Spain; 5 Red Cross University School of Nursing, Avda. de la Cruz Roja 1 Street, 41009 Seville, Spain; 6 Research Group CTS1137: Neurological Physiotherapy, Innovative Neurorehabilitation and Neurodevelopment Disorders, Avenzoar 8 Street, 41008 Seville, Spain; ignsolmar@gmail.com

**Keywords:** amyotrophic lateral sclerosis, pulmonary volume measurements, cough, daily activities, respiratory therapy

## Abstract

Background: Respiratory complications in patients with amyotrophic lateral sclerosis (ALS), due to the involvement of respiratory muscles, are the leading cause of death, and respiratory physiotherapy (RP) focuses on addressing these complications. Objectives: The objective was to evaluate the effectiveness of an RP intervention that combines the four specific techniques (inspiratory muscle training, lung volume recruitment, manually assisted coughing, and diaphragmatic breathing training) in patients with ALS. Methods: A quasi-experimental study was carried out, and a specific RP programme was implemented in 15 patients with ALS (12 sessions, 30 min/session, one session/week, duration of three months), based on directed ventilation techniques, lung volume recruitment, manually assisted coughing, and the use of incentive spirometry and a cough assist device, along with a daily home exercise programme. Respiratory functions were assessed (pre- and post-intervention, with follow-up at three months) using Forced Vital Capacity (FVC) and Peak Expiratory Cough Flow (PECF); functionality was assessed using the Revised ALS Functional Rating Scale (ALSFRS-R) and the Modified Barthel Index by Granger. Results: FVC experienced an increase after three months of the intervention initiation (*p* = 0.30), which was not sustained at the three-month follow-up after the intervention ended. All other variables remained practically constant after treatment, with their values decreasing at follow-up. Conclusion: A specific RP intervention could have beneficial effects on respiratory functions, potentially preventing pulmonary infections and hospitalisations in patients with ALS. It may improve FVC and help stabilize the patient's functional decline. Considering the progressive and degenerative nature of the disease, this finding could support the usefulness of these techniques in maintaining respiratory function.

## 1. Introduction

Amyotrophic lateral sclerosis (ALS) is a degenerative neurological disease that affects the pyramidal pathway in the first and second motor neurons [1]. However, there is evidence supporting the designation of ALS as a syndrome characterised by the degeneration of both motor neurons, which share characteristic clinical symptoms, rather than a single disease [2]. Currently, its aetiology is considered multifactorial, involving a combination of genetic factors as well as environmental and lifestyle factors [3,4]. The cause of most ALS cases remains unknown, although over 50 genes have been linked to the condition, predominantly involving missense mutations. Significant genes include SOD1, SETX, TARDBP, FUS, OPTN, UBQLN2, PFN1, TBK1, and NEK1, as well as the hexanucleotide repeat mutation in C9ORF72. In familial cases of ALS, which represent around 5% of total instances, these mutations account for 25–35% of cases. In contrast, for sporadic ALS, which constitutes the majority of cases, they contribute only 5–10% of cases [5,6,7].

The global incidence of ALS is estimated to be 1.5 to 2.7 per 100,000 person-years, with prevalence rates ranging from 4 to 10 per 100,000 people. The annual incidence of this disease in Europe is 1.8 cases per 100,000 inhabitants per year. In Spain, this figure was 4 cases per 100,000 inhabitants per year (1.6 men vs. 1.2 women) [8,9]. Other studies also indicate that ALS is more common in men, with a male-to-female ratio of approximately 1.3:1. The onset of ALS typically occurs between the ages of 50 and 70 years, and the disease is invariably progressive [10,11].

The average life expectancy is typically from three to five years after diagnosis, with death usually resulting from respiratory complications such as aspiration, pulmonary infections, and respiratory failure [12]. These difficulties arise from the progressive weakness of the respiratory muscles, which affects pulmonary function and effective cough mechanisms [13].

Therefore, early intervention, which addresses respiratory complications correctly, is essential in these patients due to the rapid progression of the disease [14]. Particularly, patients diagnosed with multidimensional, progressive ALS with a severe prognosis require interdisciplinary intervention, both in hospital and home care settings [15,16].

Spirometry is a basic test for studying pulmonary function, and its utility extends beyond the field of pulmonology; it is being progressively incorporated into other medical disciplines. It is a rapid, simple, and non-invasive test that provides important parameters about lung function. Among these parameters is FVC, defined as the maximum amount of air exhaled forcefully starting from a full inhalation. This parameter is widely used and studied in ALS because it is sensitive to pulmonary function deterioration and capable of detecting hypoventilation [17,18]. Its value reflects the functionality of inspiratory and expiratory muscles and depends on the strength of these muscles, as well as the individual’s sex, weight, age, and ethnicity. Values ≥ 80% are considered normal. Values below 50% indicate the onset of respiratory failure [19,20,21].

Another important aspect to consider in these patients is coughing, whose efficacy depends on the integrity of bulbar centres and inspiratory and expiratory muscles. Its deterioration leads to the accumulation of secretions and, consequently, a higher probability of respiratory infections [22]. Measurement of cough capacity is carried out with instruments that measure peak cough flow (PCF), defined as the maximum airflow generated during a cough. Values greater than or equal to 270 L/min indicate proper pulmonary function, while values < 160 L/min indicate ineffective coughing [23,24].

Respiratory physiotherapy (RP) is essential for both the functional recovery of patients with ALS and for reducing complications and their potential impact. In a systematic review by Macpherson and Bassile (2016), RP interventions were divided into four groups: (1) inspiratory muscle training, (2) lung volume recruitment, (3) manually assisted coughing, and (4) diaphragmatic breathing training. However, due to the progressive muscle weakness in ALS patients, the effectiveness of RP, as well as the combination of different interventions, is not clear [25].

To date, we are not aware of any studies that have evaluated the effectiveness of an RP intervention combining techniques from all four mentioned groups, despite the potential benefits this combination may offer to patients. Therefore, the aim of the present study is to evaluate the effectiveness of an RP intervention that combines the four specific techniques (inspiratory muscle training, lung volume recruitment, manually assisted coughing, and diaphragmatic breathing training) in patients with ALS.

## 2. Materials and Methods

### 2.1. Experimental Design and Sample Size Calculation

A prospective study based on a quasi-experimental design was conducted. Participants were consecutively recruited by a nurse (RCM) at the Virgen Macarena University Hospital (Seville, Spain) during 2018 and 2019.

The sample size calculation was performed using the G*Power software version 3.1.9.7. (Heinrich-Heine-Universität Düsseldorf, Düsseldorf, Germany), following guidelines for small samples. It was considered that the population of patients with ALS treated at the hospital was a total of 45 individuals, with a statistical power greater than 80% and a statistical significance level of 95%, establishing a necessary sample size of 15 participants.

An evaluating physiotherapist (AH) screened which patients met the following inclusion criteria: (1) diagnosis of ALS, established by the revised El Escorial criteria, including those classified as definite or probable, and (2) ability and willingness to comply with study procedures and follow-up requirements. Subjects who were smokers, had a history of lung pathology, significant risk of death, and/or frontotemporal dementia were excluded from the study [26].

### 2.2. Intervention

The RP intervention was carried out by a treating physiotherapist (EM), with years of clinical experience in treating respiratory complications in neurological patients. Each subject received a 30 min session once a week for three months, totalling 12 sessions. Additionally, each subject was required to perform the same protocol at home once a day with the assistance of the primary caregiver. During the first session, the participant and caregiver were trained by the physiotherapist on how to perform the exercises. To monitor adherence to the programme, a tracking diary was provided to each subject, which they had to submit to the physiotherapist weekly.

The intervention focused on preserving lung function and proper secretion drainage, employing the following techniques [27,28]: (1) directed ventilation, (2) incentive spirometry, (3) manually assisted cough techniques, and (4) mechanical cough assist.

The directed ventilation technique consisted of chest expansion exercises and diaphragmatic breathing to promote proper expansion of the chest cavity and preserve diaphragm activation in patients where it is not yet compromised. Participants performed a series of five repetitions for each exercise, with a three-minute rest period between them. The exercises based on the incentive spirometer consisted of three sets of 15 repetitions, with a two-minute rest period between each set.

The manually assisted cough techniques at the abdominal level were performed before recruiting the lung volumes with the Ambu bag, with five repetitions in one set. Finally, a mechanical cough assist device (Cough Assist E70 by Philips Respironics^®^, Phillips, Germany) was used, performing three sets of five repetitions of the inspiration/exhalation cycle (pressure range −30/+30; 15 Hz oscillation). Subsequently, a series of manually assisted coughs was performed, using the same technique as mentioned earlier, with a three-minute rest period between each set.

### 2.3. Variables and Data Collection

The patients were assessed by an evaluating physiotherapist (AH) at three evaluation points: pre-intervention, post-intervention (three months after the start of the intervention), and at three months after the intervention ended (six months after the intervention began). Sociodemographic variables such as sex, age, disease duration, type of ALS, and BiPAP device (Bilevel Positive Airway Pressure) use were collected.

Primary variables were collected to assess lung function using spirometry (Spirometer Spiroperfect by Welch Allyn^®^, Baxter Hillrom Welch Allyn, United Kingdom) [29], in terms of FVC, and cough effectiveness expressed through PCF measured with a PeakFlow device (Datospir Peak 10^®^, Sibelmed, Spain) [30].

On the other hand, a series of secondary variables were collected measured by different scales and indices.

Functionality was assessed using the Revised ALS Functional Rating Scale (ALSFRS-R). This scale consists of 12 questions that explore the three domains of impairment: bulbar, spinal, and respiratory. Each item can score from zero (maximum disability) to four (normal function), with the maximum score of the scale being 48, corresponding to normal functionality [31].

The degree of autonomy was assessed using the Modified Barthel Index by Granger. This scale consists of 15 items divided into two different subscales (self-care and mobility) and explores the level of independence in performing activities of daily living. Each item has three scoring ranges: independence, with assistance, and dependence. The maximum score achievable is 100, corresponding to the patient’s independence [32].

### 2.4. Statistical Analysis

Descriptive statistics were used to map and summarise the characteristics of the sample. Continuous variables were expressed as mean values ($\bar{x}$) with a confidence interval (CI) and categorical variables as percentages (%) and CI. The comparison of the different groups’ proportions was assessed using the χ^2^ test for categorical variables and Student’s t-test for continuous variables. Significance was considered at 0.05, and normality was evaluated using the Kolmogorov–Smirnov test.

The effect of assessment timing (i.e., pre-treatment, post-treatment, and at three months post-treatment) on the different clinical variables collected was analysed using repeated-measures ANOVAs. The inclusion of the “Granger score” factor in the model allowed for the examination of interactions between this score and the temporal changes in clinical outcomes, adding depth to the analysis. The f-values, degrees of freedom, and *p*-values are presented in Table 1 to indicate the statistical significance of the model and each factor tested. Partial eta-squared (π^2^p) values were reported as an indicator of effect size, as they provide an understanding of the magnitude of the effect observed, beyond just statistical significance.

Values between 0.1 and 0.3 were considered moderate effects, while values above 0.3 indicated substantial or important effects. Pairwise comparisons for significant models were subsequently performed using Holm–Bonferroni corrections, a conservative method to adjust for multiple comparisons and reduce the risk of type I errors. Group scores at different assessment time points were depicted in boxplot graphs that include the 25th, 50th (median), and 75th percentiles, as well as means and standard errors. Asterisks indicate significant post hoc tests (* *p* < 0.05; ** *p* < 0.01).

All statistical analyses and visualisations were conducted using R Studio software version 4.0.3 (R Studio Inc., Boston, MA, USA), a widely accepted statistical platform that allows for reproducibility and transparency in data analysis.

### 2.5. Ethical Aspects

This study was approved by the Ethics Committee for Research (CEIC) of the Virgen Macarena–Virgen del Rocío University Hospitals, with code 1660, and was conducted at the Virgen Macarena University Hospital. All participants provided written informed consent, and the study was conducted following the ethical recommendations of the most recent version of the 1975 World Medical Association Declaration of Helsinki. The manuscript followed the Sex and Gender Equity in Research (SAGER) guidelines.

## 3. Results

### 3.1. Characteristics of the Participants

The study group consisted of 15 patients (12 men vs. 3 women), aged between 38 and 76 years, with a mean age of 55.1 ± 11.0 years and a disease duration of 10.5 ± 8.2 months. Overall, 53.6% (*n* = 8) of the participants had bulbar-onset ALS, while the rest (*n* = 7) had spinal-onset ALS. Finally, 40% (*n* = 6) of the participants were users of a BiPAP device. All BiPAP device users had bulbar-onset ALS and an FVC less than 50% of the predicted value [33].

### 3.2. Results of the Intervention

Table 1 presents the results of the analysis of variance (ANOVA) for mixed linear models assessing the effect of the moment of assessment (pre-treatment, post-treatment, and at three months post-treatment), Granger score, and their interaction on various clinical variables: FVC, PCEF, ALSFRS-R, and the Granger score itself.

For FVC, there was a statistically significant main effect of the moment of assessment on FVC (F = 5.47, *p* = 0.02, π^2^p = 0.30), indicating that the FVC significantly changed across the different time points. The partial eta-squared value (π^2^p) of 0.30 suggests a moderate effect size. The Granger score did not significantly affect FVC (F = 0.08, *p* = 0.77, π^2^p = 0.01), suggesting no meaningful contribution of the Granger score to FVC changes. The interaction between the moment of assessment and the Granger score was not statistically significant (F = 1.2, *p* = 0.31, π^2^p = 0.08), indicating that the effect of the assessment time on FVC did not vary significantly based on the Granger score.

Regarding PCEF, a significant effect of the moment of assessment was observed for PCEF (F = 3.51, *p* = 0.04, π^2^p = 0.21), showing that PCEF varied significantly across the different time points. The effect size (π^2^p = 0.21) indicates a moderate impact. No significant effect was found for the Granger score on PCEF (F = 0.08, *p* = 0.78, π^2^p = 0.01), implying that the Granger score did not influence changes in PCEF. The interaction effect was also not significant (F = 1.93, *p* = 0.16, π^2^p = 0.13), meaning the Granger score did not modify the influence of assessment time on PCEF.

For ALSFRS-R, no statistically significant differences were found in ALSFRS-R across time points (F = 2.17, *p* = 0.13, π^2^p = 0.14), suggesting that the ALS functional score remained relatively stable throughout the study. The partial eta-squared value (π^2^p = 0.14) indicates a small effect. The Granger score did not significantly affect ALSFRS-R scores (F = 1.37, *p* = 0.26, π^2^p = 0.09). The interaction was not significant (F = 1.82, *p* = 0.18, π^2^p = 0.12), indicating that the effect of assessment time on ALSFRS-R was not influenced by the Granger score. The Granger score was significantly affected by the time of assessment (F = 3.4, *p* = 0.04, π^2^p = 0.20), indicating that it changed over time with a moderate effect size.

Finally, for the Granger score, there was a highly significant main effect for the Granger score itself (F = 11.9, *p* < 0.01, π^2^p = 0.47), suggesting a substantial effect. The interaction between the moment of assessment and the Granger score was not significant (F = 0.33, *p* = 0.72, π^2^p = 0.02), indicating no interaction between the time of assessment and the Granger score in this case.

**Figure 1:**
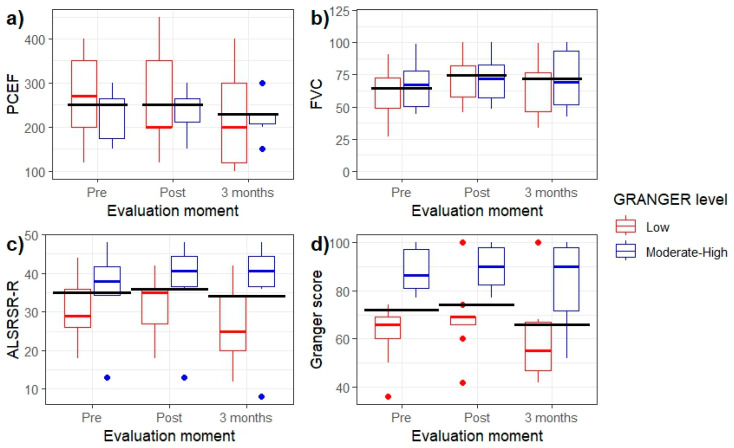
Box plots illustrating scores on the PCEF (**a**), FVC (**b**), ALSRSR-R (**c**), and Granger score (**d**) scales across the three evaluation moments for low (red) and moderate-high (blue) Granger levels). Based on the Granger scale’s scores, the sample was divided into two level: patients with a Granger score less than 60 (low) and patients with a Granger score greater than 60 (moderate–high) [32]. Black horizontal bars indicate median values for the entire group (i.e., without stratification by Granger level).

**Figure 2:**
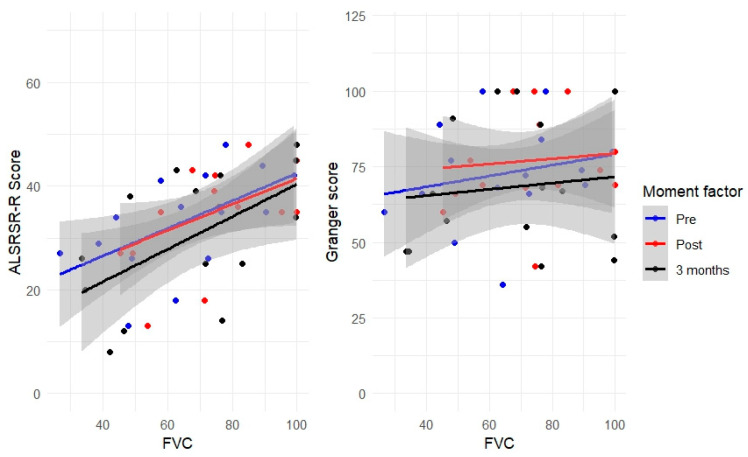
Scatter plot of the scores from the scales used.

The patients’ scores at different evaluation time points are illustrated in Figure 1. The analyses revealed significant effects of the evaluation time on the FVC, PCEF, and Granger values. Post hoc non-parametric analyses revealed a generalised decrease in PCEF after 3 months of follow-up compared to both pre- and post-treatment time points. No differences were observed between these latter two time points. Regarding the FVC scale, there was an increase in scores after treatment compared to before treatment. However, at 3 months, the scores were comparable to those obtained in the other two evaluations. Finally, significant differences were observed between post-treatment and the 3-month scores on the Granger scale (Figure 2).

## 4. Discussion

In this quasi-experimental study, the effects of an RP intervention based on the use of incentive spirometer, cough assist, and directed ventilation techniques, as well as lung volume recruitment and assisted coughing, are analysed. Among the results obtained, a significant improvement in FVC measured before and after the intervention is highlighted. The other measured variables show no changes post-intervention, remaining practically constant. All measured variables, both those related to lung function and those related to functionality (ALSFRS-R and the Granger Index), remained practically constant throughout the entire study. Palliative-care PR appears to mitigate the slope of the ALSFRS-R, and prolonged disruption of rehabilitation during confinement may have accelerated the functional decline of motor skills in ALS patients, as measured after 2 months by the ALSFRS-R, increasing fatigue and negatively impacting quality of life [34]. Despite not identifying a clear improvement after the intervention, the absence of worsening, given the progressive and degenerative characteristics of the disease, could justify the need for this type of intervention [5]. Furthermore, some authors recommend metrics that assess respiratory function independently of the ALSFRS-R [35]. This is especially reflected in the improvement in FVC, as is also the case with interventions based on respiratory muscle training [25].

Although telerehabilitation can contribute to better monitoring of ALS patients, some barriers have been detected, both in patients/caregivers and in healthcare professionals, such as lack of physical evaluation/contact, uncertainty about comprehensive medical assessment, and no time saving/costing extra time [36]. Thus, the need to investigate respiratory techniques [37] that, although they must be performed in person by the physiotherapist, do not entail excessive economic or time costs for healthcare systems becomes evident. One of the advantages of the devices used in PR is that they can be taken home, and as evidenced by the nurses’ visits, the need for physiotherapy and teaching the precise manner for airway clearance to family caregivers is evident [38].

The intervention protocol carried out in this study was designed considering that the main cause of death in these patients is respiratory complications [12]. In this regard, this combination of various RP techniques, most in line with the existing literature arguing that inspiratory muscle training, lung volume recruitment training, and manually assisted cough have effectiveness in improving respiratory outcome measures and increasing survival [25]. In the present study, an incentive spirometer and cough assist were used as tools to promote lung expansion; this implies that techniques were included to promote lung expansion, used to maintain proper recruitment of lung volumes, and proper activation of the involved musculature (as therapeutic training programmes that include inspiratory and expiratory force targets have been shown to optimise airway clearance ability in this challenging patient population [39] as well as techniques for secretion removal, which are the main culprits of respiratory infections [22]. This represents a further step in RP compared to other interventions previously evaluated, based on mechanical insufflation/exsufflation exercises aimed at increasing peak cough flow [40].

It is worth considering the fact that the use of the incentive spirometer presupposes activation of the inspiratory musculature, which, although it does not involve working with a load/resistance as typically trained in this musculature [25], can lead to its activation, maintenance, and even training. The effect of different types of muscle strength training has not been widely studied in ALS patients [41], and their impact on fatigue is unclear [42]. Additionally, the use of assisted coughing preceded by hyperinflation with an Ambu bag and the use of the cough assist as a tool to promote lung expansion and at the same time remove secretions could contribute to the improvement and maintenance of FVC. The fact of including this intervention in the protocol followed by the patients in the present study would be justified in the literature, since lung volume recruitment treatment could increase control over airway clearance in ALS, offering patients control over some of the most feared symptoms of ALS, particularly choking during activities of daily living, and enhanced ALS respiratory care in low-resource settings [43]. This has the advantage of reducing the constant accumulation of secretions and thus promoting proper lung expansion [28].

Regarding manually assisted coughing techniques, classified by the ICF under additional respiratory functions, they have been identified in reviews such as those by Cleary et al. (2021) [43] and Macpherson and Bassile (2016) [25] as techniques that improve lung volume recruitment function when evaluated just before and after execution. The clinical utility of these techniques is also evident from findings in the study by Cleary et al. (2013) [44], where FVC was measured before and after executing five hyperinflations followed by two trials of assisted coughs, recording an increase in this variable at 15 min post-execution. These techniques may thus have a positive effect on FVC by promoting rapid and immediate secretion removal. Furthermore, according to a subsequent study by the same author, these techniques are easy to use, cost-effective, and simple to learn for both patients and caregivers, allowing them to be performed daily at home as a tool against secretion accumulation, providing a sense of control and security [43].

Support of cough function with manual assistance, a resuscitator bag, and/or mechanical insufflation–exsufflation can help prevent and treat infection [45]. In addition, the use of the cough assist device could have been an added advantage to the intervention due to its efficacy in clearing secretions, with good patient tolerance, offering the possibility of daily use at home by the patient or assisted by the caregiver, with prior training, as evidenced by Bento et al. [46]. Its daily use, even in patients without impaired cough capacity, could contribute to the improvement of respiratory functions, despite authors such as Paneroni et al. (2014) finding in their home-based study that there were no differences in outcomes when an intervention based on coughing was conducted [47]. Based on these results, its use could be justified in all patients from the diagnosis of the disease, in order to promote lung expansion, due to the insufflation pressure. This device is used as a non-invasive mechanically assisted cough technique, based on an insufflation–exsufflation mechanism, whereby positive pressure is applied to the airways, quickly followed by negative pressure, which achieves airway clearance [39]. Thus, these techniques can be used from the onset of the disease, facilitating respiratory function, with the aim of preventing complications and improving quality of life [43], but especially to gradually accustom the patient to its use, mastering it in the future when dependence on it arises due to ineffective coughing in clearing secretions.

Regarding functionality, our results are in line with those found in other previous studies [25,48]. After the intervention, there was no increase in the different spheres of impairment measured by the ALSFRS-R. Nor did the scores obtained from the Modified Barthel Index by Granger show improvement.

The duration of the intervention represents another novel contribution to this study. As highlighted in the review by Macpherson and Basile (2016) [25], studies involving a longer intervention period (e.g., 12 weeks or 8 months) only focus on inspiratory muscle training, without considering other aspects such as coughing which are associated with the clinical presentation of these patients. In this sense, comparing our overall results with those obtained in other previous studies, only interventions that had been carried out using respiratory muscle training contributed to an improvement in FVC [49,50].

On the other hand, there exists a scarcity of studies of specific interventions based on inspiratory and expiratory muscle training with no improvement in FVC or ALSFRS-R, as highlighted by the recent meta-analysis by Su et al. (2021) [48] on the effects of RP programmes. This implies that there is still no solid evidence on the efficacy of inspiratory muscle training in these patients. One of the instruments that seems to improve lung expansion and activation of the inspiratory muscles is training using the incentive spirometer, a device widely used in patients who have undergone lung and cardiothoracic surgery, whose use is also indicated in patients with neuromuscular pathologies [51]. Despite this, RP protocols including it for ALS patients have not been reported.

For this reason, and as prospective lines, we consider it necessary to develop a clinical trial to compare the intervention group with a control group to try to obtain more enlightening data on the RP intervention described in this study. The lack of a control group is also a limitation in this study, which requires us to interpret the results found with caution. Another limitation of the present study is the limited sample size, which makes it difficult for us to extrapolate the results found, even though the study population, given the prevalence of the disease, is also very small. Finally, it is worth noting as a limitation that the ALS genotype of the included patients has not been considered. In this regard, various studies suggest that individuals with altered genes such as SOD1 and C9ORF72 have a higher likelihood of developing respiratory insufficiency than those with mutations in other genes related to this disease [52].

## 5. Conclusions

The respiratory physiotherapy intervention based on a combination of techniques (inspiratory muscle training, lung volume recruitment, manually assisted coughing, and diaphragmatic breathing training) aimed at promoting lung expansion and proper secretion drainage has improved Forced Vital Capacity values. All other measured variables remained practically unchanged after treatment, with their values decreasing at follow-up. The fact that they remained stable throughout the study, despite the progressive and degenerative nature of the disease, could support the effectiveness of these techniques in maintaining respiratory functions. The length of the entire intervention and the combination of the four respiratory physiotherapy techniques (mentioned before) in a session represent the innovative contribution of this study. Although the results should be interpreted cautiously due to the small sample size, this study offers valuable suggestions to take into account for clinical practice. First, it highlights the importance of providing care focused on long-term respiratory training under the supervision of a specialised physiotherapist with extensive training in respiratory management of neurological patients. This professional not only treats the patient directly but can also educate and train the primary caregiver to ensure the continuity of care. Finally, the proposed intervention is simple to implement, requires minimal time per session, and is cost-effective.

## Figures and Tables

**Table 1:** Analysis of variance of mixed linear models.

	Moment of Assessment			Granger Score			Moment of Assessment * Granger Score		
	F (*df*1, *df*2)	*p-*Value	π^2^p	F (*df*1, *df*2)	*p-*Value	π^2^p	F (*df*1, *df*2)	*p-*Value	π^2^p
**FVC**	5.47 (2.28)	0.02	0.30	0.08 (1.13)	0.77	0.01	1.2 (2.28)	0.31	0.08
**PCEF**	3.51 (2.28)	0.04	0.21	0.08 (1.13)	0.78	0.01	1.93 (2.28)	0.16	0.13
**ALSFRS-R**	2.17 (2.28)	0.13	0.14	1.37 (1.13)	0.26	0.09	1.82 (2.28)	0.18	0.12
**GRANGER**	3.4 (2.28)	0.04	0.20	11.9 (1.13)	<0.01	0.47	0.33 (2.28)	0.72	0.02

* *p* < 0.05. ALSFRS-R: Revised ALS Functional Rating Scale; FVC: Forced Vital Capacity; PCEF: Peak Expiratory Cough Flow.

## Data Availability

Data available on request.
